# Effects of* Xingpi Kaiyu Fang* on ATP, Na/K-ATPase, and Respiratory Chain Complexes of Hippocampus and Gastrocnemius Muscle in Depressed Rats

**DOI:** 10.1155/2019/6054926

**Published:** 2019-01-03

**Authors:** Qingjie Yuan, Yang Li, Xiaofei Deng, Huawei Shi, Zhenwu Zhao, Chunye Wang, Xuegong Feng, Jianyou Guo, Rongjuan Guo

**Affiliations:** ^1^Beijing Hospital of Integrated Traditional Chinese and Western Medicine, Beijing 100039, China; ^2^Beijing University of Chinese Medicine, Beijing 100029, China; ^3^CAS Key Laboratory of Mental Health, Institute of Psychology, Chinese Academy of Sciences, Beijing 100101, China; ^4^Dongfang Hospital Beijing University of Chinese Medicine, Beijing 100078, China

## Abstract

**Objective:**

To clarify the effectiveness and mechanism of the Chinese herbal formula* Xingpi Kaiyu Fang* (XPKYF) which is composed of* American ginseng* (Xi-Yang-shen),* Radix curcumae* (Yu-Jin),* Acori tatarinowii rhizoma* (Shi-Chang-pu), and* Hypericum perforatum* (Guan-Ye-lian-qiao) in depressed rats.

**Methods:**

The rat model of depression was established by chronic unpredictable mild stress (CUMS) method for 6 weeks. Rats were randomly divided into six groups: control group, CUMS group, CUMS+XPKYF (3.6g/kg/d, 7.2g/kg/d, 14.4g/kg/d) groups, and CUMS+sertraline (4.5mg/kg/d) group. The sucrose preference test and the forced swimming test were performed to assess the rats' depression behavior. Mitochondrial ultrastructure was observed by transmission electron microscope and adenosine triphosphate (ATP) content, sodium potassium ATPase (Na/K-ATPase) activity, and mitochondrial respiratory chain complexes activities in hippocampus and gastrocnemius muscle were measured at the 14^th^ and 42^nd^ day.

**Results:**

Rats subjected to six weeks of CUMS exhibited decreased sucrose preference ratio and prolonged immobility time. CUMS reduced ATP content in hippocampus, decreased Na/K-ATPase activity and respiratory chain complex I, III, and IV activities in hippocampus and gastrocnemius muscle, and damaged mitochondrial ultrastructure of hippocampus and gastrocnemius muscle. XPKYF at 14.4g/kg, the efficacy trend of which was better than the other drug groups, could prevent the stress-induced depressed behavior changes, inhibit the decrease of Na/K-ATPase activity in hippocampus, inhibit the decrease of respiratory chain complex III activities in hippocampus and gastrocnemius muscle, and protect mitochondria from ultrastructural damage.

**Conclusions:**

Energy deficiency and damaged mitochondrial ultrastructure were found in hippocampus and gastrocnemius muscle of depressed rats established by CUMS. XPKYF could partly reverse alterations in ATP, Na/K-ATPase, and respiratory chain complexes of hippocampus and gastrocnemius muscle and protect mitochondria from ultrastructural damage. This provides another experimental evidence for the clinical application of XPKYF in the treatment of depression.

## 1. Introduction

Depression is a complex disease, clinically manifested by multisystem damage symptoms involving many mental and physical symptoms [1], seriously affecting patients' health. More than 350 million people are affected by depression; as the biggest cause of disability, approximately two-thirds of those who commit suicide have the condition [2]. Up to one-third of depressive patients show poor responses to conventional antidepressants [3] at present, of which selective serotonin reuptake inhibitors account for the major proportion, and furthermore a delay in symptom remission [4] and severe side effects [5] commonly occur during the administration of these drugs. Therefore, depression is still an important issue worthy of more attention especially on the aspect of discovering effective therapies.

Mitochondria are the power plants of life activities in all eukaryotic cells, and the organs or tissues rich in mitochondria, such as hippocampus and skeleton muscles, are more susceptible to mitochondrial dysfunction. Hippocampus is a vital brain region related to mental activity and memory, and loss of hippocampal volume is considered to have close relationship with depression, and an increase in hippocampal grey matter was observed following administration of antidepressant [6]. Skeletal muscle is an important part of moving system, and the dysfunction in skeletal muscle can result in fatigue, a common symptom in depression. In recent years, mounting evidences have shown that not only the mitochondrial respiratory rate of platelets and mononuclear cells in peripheral blood of depressed patients, but also the ATP production in muscle tissues of depressed patients with somatization symptoms had been found decreased [7–9], which even to some extent indicates that the energy metabolism dysfunction in depression is not only confined to the central nervous system, but also related to the peripheral system.

Xingpi Kaiyu Fang (XPKYF), a Chinese herbal formula, is composed of four herbs as follows:* American ginseng (Xi-Yang-shen), Radix curcumae (Yu-Jin), Acori tatarinowii rhizoma (Shi-Chang-pu), and Hypericum perforatum (Guan-Ye-lian-qiao)*. It derived from the improvement on Xingpi Jieyu (XPJY) decoction by replacing the* Radix rehmanniae proparate* with the* Hypericum perforatum*. Consistent with our clinical practices, similar to antidepressant medication, the* Hypericum perforatum* can better improve depression symptoms in mild and moderate depression than placebo [10]. Previous studies showed that XPJY decoction had an antidepressant effect in rats exposed to chronic stress through increasing serum serotonin, decreasing serum corticosterone, reducing the inflammatory factors in serum and hippocampus, and up-regulating cAMP-PKA-CREB-BDNF pathway in hippocampus [11–13]. In accordance with these above data, XPKYF also showed a similar significant effect on inhibiting stress-induced endocrine dysfunction and inflammatory disorders in our preliminary study [14]. At present, XPKYF has already got the patent, and the patent number is 201510047421.4. However, whether it can affect energy level and mitochondria remains to be further clarified. Adenosine triphosphate (ATP) is the major energy sources of all eukaryotic cells. Its production requires four respiratory chain complexes located in mitochondrial inner membrane as follows: complex I (NADH-ubiquinone oxidoreductase), complex II (succinate-quinone oxidoreductase), complex III (cytochrome bc_1_ complex), and complex IV (cytochrome c oxidase). Sodium potassium ATPase (Na/K-ATPase), an ion pump drove by ATP, widely distributed in the plasma membrane of all animal cells, is vital for cellular function such as cell growth, differentiation, migration, contraction, secretion, and volume regulation [15]. ATP, Na/K-ATPase, and mitochondrial enzyme complexes all can reflect the function of mitochondria, so the present study was to explore the mitochondrial impairment characteristics and to clarify the intervening effects of XPKYF on ATP, Na/K-ATPase, and respiratory chain complexes of hippocampus and gastrocnemius muscle in rats exposed to chronic unpredictable mild stress (CUMS).

## 2. Materials and Methods

### 2.1. Preparation and Compositional Analysis of XPKYF

**Figure 1:**
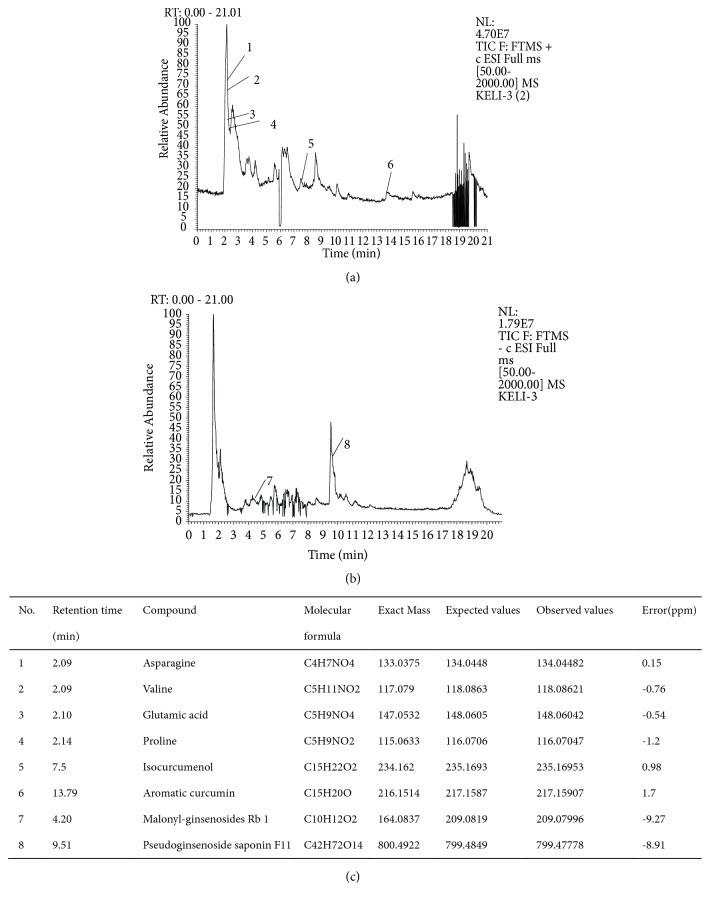
Total ion chromatography. (a) Total positive ions chromatography. (b) Total negative ions chromatography. (c) Results of ions identification.

XPKYF was composed of the following granules, which derived from dried raw materials, including* American ginseng (Xi-Yang-shen), Radix curcumae (Yu-Jin), Acori tatarinowii rhizoma (Shi-Chang-pu), and Hypericum perforatum (Guan-Ye-lian-qiao)*, in weight ratio of 1:1:1:1. All granules were bought from medicinal materials company of Beijing KangRenTang Company (Beijing, China) and analyzed for composition by high resolution mass spectrometry (HRMS). Instrument type: Thermo LTQ-Orbitrap hybrid mass. Chromatographic conditions: column temperature (30°C), flow rate (0.03ml/min), chromatographic column: Thermo scientific 150 × 2.1mm BDSHYPERSIL C18. mobile phase: A: 0.1% formic acid aqueous solution, B: acetonitrile. Elution procedure: 0-0.3min 5%B, 0.3-0.6min 5%-12%B, 0.6-7min 12%-28%B, 7-15min 28%-32%B, 15-18min 32%-98% B. Mass spectrometry conditions: ion mode: positive ion mode; heater temperature (35°C). capillary temperature (35°C), capillary voltage (35kv), sheath gas velocity (35arb), auxiliary gas velocity (10arb). Quality scanning range: 50-2000, resolution: 30000. Sample treatment method: precise weighed 3.09g particles, placed in a cone flask, precision measurement of 50% methanol solution 60 ml added. Ultrasonic 30 minutes. Take the filtrate 1 ml, through the 0.45um micro film, to prepare sample solution (Figure 1).

### 2.2. Animals and Grouping

Adult male Wistar rats, weighting 160~180g, were provided by Beijing Vital River Laboratory Animal Technology Co., Ltd. These rats were housed in individual cages under a 12 h/12h light/dark cycle and at room temperature. Food and water were made available ad libitum. Rats were acclimated to 3 min of handling once a day for 7 consecutive days before being used in experiment. All animal procedures conformed to the Guide for the Care and Use of Laboratory Animals.

One hundred and eight rats were assigned randomly into six groups: control, CUMS, CUMS + XPKYF 3.6g/kg/d, CUMS + XPKYF 7.2g/kg/d, CUMS + XPKYF 14.4g/kg/d, and CUMS + sertraline 4.5mg/kg/d. There were eighteen rats in each group. Rats in control group received once daily intragastric administration of saline for 42 days; rats in CUMS group were exposed to unpredictable stressors and received once daily intragastric administration of saline for 42 days; rats in XPKYF groups were exposed to unpredictable stressors and received once daily intragastric administration of XPKYF at 3.6g/kg/d, 7.2g/kg/d, and 14.4g/kg/d, respectively, for 42 days; rats in sertraline group were exposed to unpredictable stressors and received once daily intragastric administration of sertraline (Pfizer Inc., USA) at 4.5mg/kg/d for 42 days. The dosages of XPKYF and sertraline were selected based on the previous experimental study [16]. The sertraline and XPKYF were all dissolved in saline.

### 2.3. Animal Model Preparation and the Experimental Schedule

**Figure 2:**
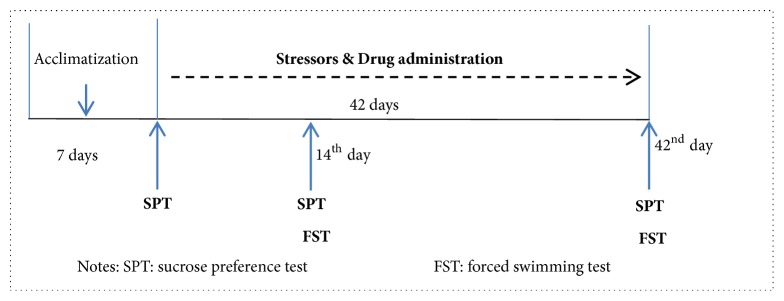
Schematic drawing of experimental schedule.

CUMS is a classical animal model of depression which can well simulate human depression that developed from suffering chronic stress. It has been developed to meet construct, face, and / or predictive validity criteria proposed for animal “depression” [17, 18]; the randomness of stress factors are the keys to the model's success. In this CUMS model, experimental animals are exposed to a series of variable stressors to induce a depression-like state [19], including anhedonia, reduced brain reward functions, and decline of sports and inquiry ability [20]. These changes in animal behaviors were assessed by decreased sucrose consumption and extension of immobility time which can well mimic the symptoms of depression and laziness/despair in human depression. In present study, the CUMS procedure was modified based on that used by Willner P [21]. Briefly, all rats excluding those in the control group were exposed to a series of multivariate stressors consisting of tilted rat cage at 30° angle, high frequency flash stimulation (200/min), 0.5h of restraining & stimulating rats on tails with forceps clip for 5 minutes, noise stimulation of rat screech, cold water swimming (4°C,5min), fasting, water deprivation, wet pad, elevated temperature in surroundings (30°C~32°C), white light irradiation, shading, and mixed breeding. 1-2 kinds of stressors were randomly exerted to the rats daily and the procedure lasted for 42 days. After one week of acclimatization, the Wistar rats were randomly divided into 6 groups. All rats except those in the control group were subjected to CUMS. XPKYF or sertraline was given through intragastric administration. The CUMS procedures were performed 30 minutes after drug administration usually by gavage between 9am and 10am. The sucrose preference tests were performed at 1^st^, 14^th^, and 42^nd^ day, and the forced swimming tests were performed at the 14^th^ day and 42^nd^ day (Table 1, Figure 2). Half of rats in each group were executed by cervical dislocation at the end of 2^nd^ and 6^th^ weeks. All procedures involving animals and their care were conducted in conformity with NIH guidelines (NIH Pub. No. 85-23, revised 1996) and were approved by Animal Care and Use Committee of Beijing University of Chinese Medicine.

### 2.4. Sucrose Preference Test

The rodents are naturally sensitive to sugar and the preference for sugar constitutes a part of reward responses. Sucrose preference test is commonly used to test rats' response to reward and the reduction of sucrose preference ratio can well simulate the core symptom of human depression “anhedonia”. The test was performed at the 1^st^, 14^th^, and 42^nd^ day. For the first time to perform the sucrose preference test, the rats were deprived of water for 24 hours at first, then a bottle with water and a bottle with 5% sucrose solution were placed to the rat cages and rats were allowed to freely consume the fluids for a period of 2 hours. Exchange the position of two bottles once in the middle time. Sucrose consumption and water consumption were measured and the sucrose preference ratio was calculated as the sucrose preference ratio (%) =sucrose consumption/(sucrose consumption + water consumption)×100%.

### 2.5. Forced Swimming Test

The immobility time in forced swimming test can reflect the despair state of rats, so it is commonly used to determine whether the rats are depressed or not. The test was performed at the 14^th^ and 42^nd^ day. Rats were placed into transparent barrels (inner diameter: 35 cm, height: 60 cm, depth of water: 35 cm, temperature of water: 25-30°C) for 5 min; then the duration of immobility time in the later 4 min was recorded. The criterion of immobility state is that the rats remain motionless or there would be only slight movements to keep the bodies from sinking.

### 2.6. Measurements of ATP Content and Na/K-ATPase Activity

At the 14^th^ and 42^nd^ day, after half of rats in each group were sacrificed, the hippocampus and gastrocnemius muscle were quickly dissected and then homogenized. Homogenate buffer (0.01 mol/L Tris, 0.0001 mol/L EDTA, 0.01 mol/L sucrose, 0.9% NaCl, pH 7.4) was added to the samples to obtain a 10% tissue homogenization. The homogenate was centrifuged at 3500 rpm for 10 minutes at 4°C, and the supernatant was collected to measure ATP content and Na/K-ATPase activity, determined by a corresponding assay kit (Nanjing Jiancheng Bioengineering Institute, Nanjing, China). The measurements of ATP content and Na/K-ATPase activity were performed as described [22].

### 2.7. Measurements of Respiratory Chain Complexes Activities

At the 14^th^ and 42^nd^ day, after half of rats in each group were sacrificed, the hippocampus and gastrocnemius muscle were quickly dissected, washed, and suspended in ice-cold isolation buffer and immediately homogenized. Homogenates were centrifuged at 1000 g, 4°C for 5 min, and the supernatant was transferred to a new tube. The pellet was resuspended in isolation buffer, homogenized, and centrifuged again. The combined supernatants were centrifuged at 8100 g for 15 min at 4°C, the mitochondrial pellet was resuspended in isolation buffer, and the aliquots were stored at −80°C. 

*Complex I (CI) Activity*. Add 10 ul mitochondrial suspension into 2 mL reaction buffer of the CI[10 mmol/L Tris-HCl (pH 8.0), 80 umol/L CoQ0, 1 mg/mL BSA, 2 mmol/L NaN3, 2 *μ*g/mL anti-amycin A], then add 200 *μ*mol/L NADH into the mixture to start the reaction. The same volume of distilled water was used as a blank control. The changes of NADH absorption values at 340 nm were continuously measured within 3 min. 

*Complex II (CII) Activity*. Add 10uL mitochondrial suspension into 2 mL reaction buffer of CII[50mmol/L potassium phosphate solution (K2HPO4: KH2PO4 = 4:1, pH 7.4), 50 *μ*mol/L DCPIP,2 mmol/L NaN3, 2 ug/mL rotenone,2ug/mL anti-amycin A, 25 umol/L CoQ0], then add 20 *μ*mol/L sodium succinate into the mixture to start the reaction. The same volume of distilled water was used as a blank control. The changes of DCPIP amount reduced by FADH2 were measured continuously within 3 min. 

*Complex III (CIII) Activity*. Add 10uL mitochondrial suspension into 2 ml reaction buffer of CIII [50 mmol/L Tris-HCl, 1mmol/L EDTA, 250mmol/L sucrose, 2 mmol/L NaN3, 50 umol/L cytochrome C (oxidation type), pH7.4], then add 80 umol/L CoQ0 (reduced type) into the mixture to start the reaction. The same volume of distilled water was used as a blank control. The changes in the absorption value of cytochrome C at 550 nm were continuously measured within 3 min. 

*Complex IV (CIV) Activity*. Add 10uL mitochondrial suspension into 2 mL reaction buffer of CIV (10mmol/L Tris-HCl, 25 mmol/L sucrose, 120 mmol/L KCl, 0.025%  *β*-cracking agent, pH 7.0), then add 50 umol/L cytochrome C (reduced type) into the mixture. The same volume of distilled water was used as a blank control. The changes in the absorption value of cytochrome C at 550 nm were continuously measured within 3 min.

The isolation of mitochondria and the spectrophotometric analysis used to evaluate activities of respiratory chain complexes I–IV were performed as described [23]. All samples were determined with a corresponding assay kit (Nanjing Jiancheng Bioengineering Institute, Nanjing, China).

### 2.8. Ultrastructure Observation of Mitochondria

At the 14^th^ and 42^nd^ day, hippocampus and gastrocnemius muscle tissues fetched from three rats randomly selected in each group were used for ultrastructural observation of mitochondria. Hippocampus and gastrocnemius muscle tissues in size of 1 mm×1 mm×3 mm were separated, next following fixation with glutaraldehyde and osmium tetroxide solutions, gradient acetone dehydration, embedding, ultrathin sections preparation and dyed with 3% uranyl acetate solution, room temperature, 10 min and lead citrate dye solution at room temperature, 10 min. Lead citrate dye solution preparation method: weigh lead 6.65g, sodium 8.8g, add a proper amount of distilled water, shake for 40 min, add saturated NaOH to solution, and then add distilled water to 250 ml. To observe the mitochondrial ultrastructure of hippocampus and gastrocnemius muscle tissues by transmission electron microscope (JEM1230), the magnification of the hippocampus is ×15000 and the magnification of the gastrocnemius muscle is ×25000.

### 2.9. Statistical Analysis

Data are presented as mean ± SEM. Differences between groups were analyzed by one-way analysis of variance followed by Dunnett's multiple comparisons. The significant level was *P* value < 0.05.

## 3. Results

### 3.1. Sucrose Preference Test

**Figure 3:**
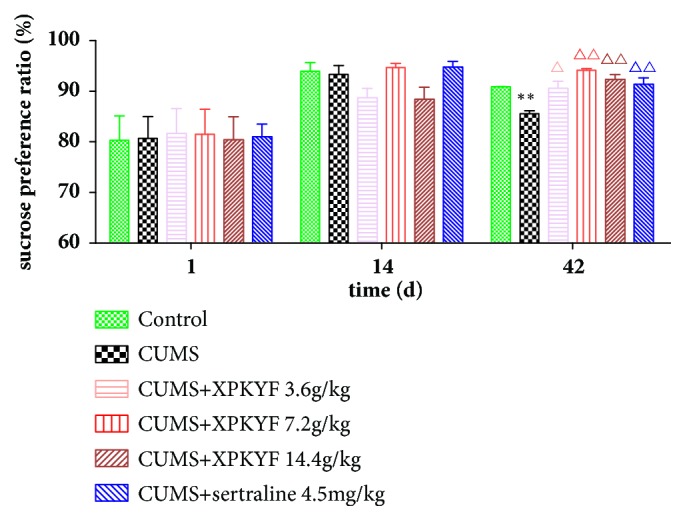
Changes of sucrose preference ratio. The CUMS procedures were performed 30 minutes after drug administration usually by gavage between 9am and 10am. Values were presented as mean ± SEM. n=18 rats per group at the 14^th^ day; n=9 rats per group at the 42^nd^ day. ^*∗*^P<0.05 versus control group; ^△^P<0.05 versus CUMS group; ^△△^P<0.01 versus CUMS group.

Rats from different groups showed no significant difference in sucrose preference ratio at the 1^st^ day and the 14^th^ day (*P* > 0.05). Significant decline of sucrose preference ratio was observed at the 42^nd^ day (*P* < 0.05) in the CUMS group. However, at the 42^nd^ day, treatment with XPKYF at 3.6g/kg, 7.2g/kg, and 14.4g/kg or sertraline at 4.5mg/kg significantly suppressed the stress-induced decline of sucrose preference ratio (*P* < 0.01 or* P* < 0.05) (Figure 3).

### 3.2. Forced Swimming Test

At the 14^th^ day, rats from different groups showed no significant difference in the immobility time (*P* > 0.05). At the 42^nd^ day, in comparison to the control group, the immobility time was significantly prolonged in CUMS group (*P* < 0.01); in comparison to the CUMS group, treatment with XPKYF at 7.2g/kg and 14.4g/kg and sertraline at 4.5mg/kg could shorten the immobility time (*P* < 0.01,* P* < 0.01,* P* < 0.05). (Table 2)

In brief, after 6-week administration of multiple variable stressors, rats in CUMS group showed a significant decline of sucrose preference ratio and prolonged immobility time. The rat model of depression was successfully established.

### 3.3. Effects of XPKYF on ATP Content

**Figure 4:**
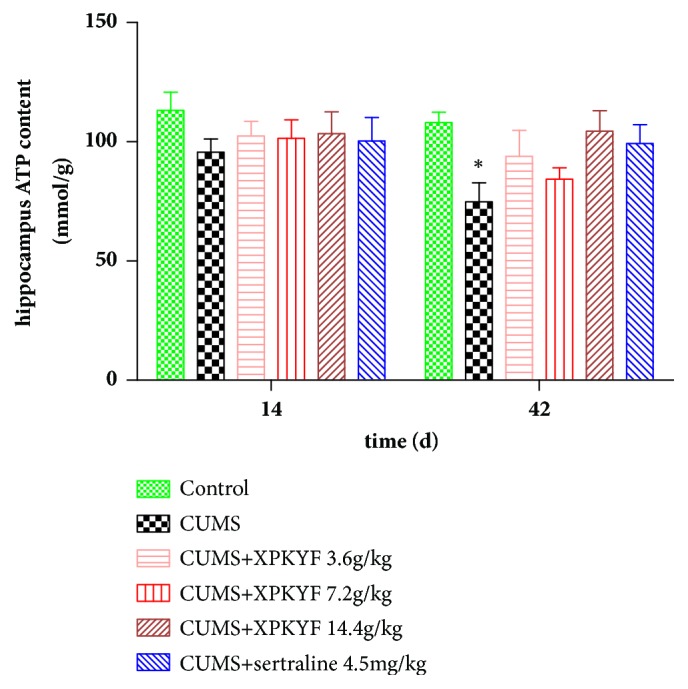
Hippocampus ATP content. The CUMS procedures were performed 30 minutes after drug administration usually by gavage between 9am and 10am. Values were presented as mean ± SEM. n=6 rats per group. ^*∗*^P<0.05 versus control group.

**Figure 5:**
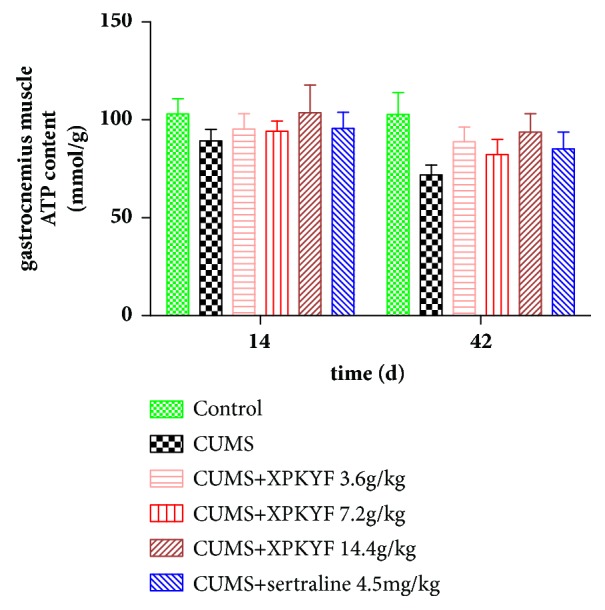
Gastrocnemius muscle ATP content. The CUMS procedures were performed 30 minutes after drug administration usually by gavage between 9am and 10am. Values were presented as mean ± SEM. n=6 rats per group.

At the 14^th^ day, rats from different groups showed no significant difference in ATP content of hippocampus and gastrocnemius muscle (*P* > 0.05). At the 42^nd^ day, in comparison to the control group, the ATP content of hippocampus significantly decreased in CUMS group (*P* < 0.05); in comparison to the CUMS group, treatment with XPKYF or sertraline could not prevent the decreased ATP content of hippocampus (*P* > 0.05) (Figures 4 and 5).

### 3.4. Effects of XPKYF on Na/K-ATPase Activity

**Figure 6:**
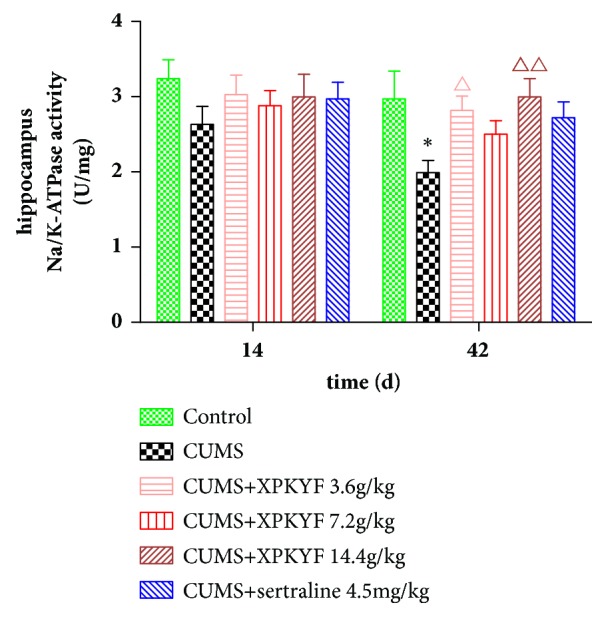
Hippocampus Na/K-ATPase activity. The CUMS procedures were performed 30 minutes after drug administration usually by gavage between 9am and 10am. Values were presented as mean ± SEM. n=6 rats per group. ^*∗*^P<0.05 versus control group; ^△^P<0.05 versus CUMS group; ^△△^P<0.01 versus CUMS group.

**Figure 7:**
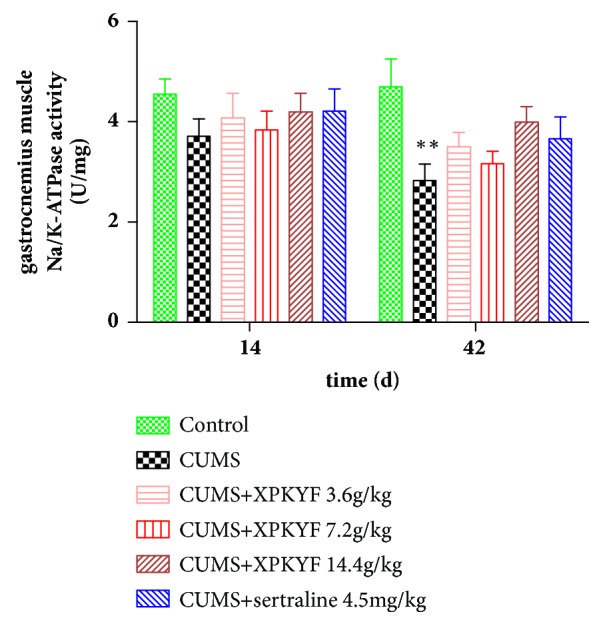
Gastrocnemius muscle Na/K-ATPase activity. The CUMS procedures were performed 30 minutes after drug administration usually by gavage between 9am and 10am. Values were presented as mean ± SEM. n=6 rats per group. ^*∗∗*^P<0.01 versus control group.

At the 14^th^ day, rats from different groups showed no significant difference in Na/K-ATPase activities of hippocampus and gastrocnemius muscle (*P* > 0.05). At the 42^nd^ day, in comparison to the control group, Na/K-ATPase activities of hippocampus and gastrocnemius muscle significantly decreased in CUMS group (*P* < 0.05,* P* < 0.01); in comparison to the CUMS group, treatment with XPKYF at 3.6g/kg and 14.4g/kg could prevent the decreased Na/K-ATPase activity of hippocampus (*P* < 0.05,* P* < 0.01) (Figures 6 and 7).

### 3.5. Effects of XPKYF on Mitochondrial Respiratory Chain Complex Activities

#### 3.5.1. Hippocampus Tissue Complex I, II, III, and IV Activities

At the 14^th^ day, rats from different groups showed no significant difference in mitochondrial respiratory chain complex II, III, and IV activities(*P* > 0.05); in comparison to the control group, mitochondrial respiratory chain complex I activity significantly declined in CUMS group(*P* < 0.05); in comparison to the CUMS group, treatment with XPKYF at 7.2g/kg and sertraline at 4.5mg/kg could suppress this decline (*P* < 0.05,* P* < 0.01). At the 42^nd^ day, rats from different groups showed no significant difference in mitochondrial respiratory chain complex II activity (*P* > 0.05); in comparison to the control group, mitochondrial respiratory chain complex I, III, and IV activities significantly declined in CUMS group (*P* < 0.05,* P* < 0.05,* P* < 0.05); in comparison to the CUMS group, treatment with XPKYF at 3.6g/kg and 14.4g/kg could suppress the decline of complex III activity (*P* < 0.05,* P* < 0.05) (Table 3).

#### 3.5.2. Gastrocnemius Muscle Tissue Complex I, II, III, and IV Activities

At the 14^th^ day, rats from different groups showed no significant difference in mitochondrial respiratory chain complex I, II, III, and IV activities (*P* > 0.05). At the 42^nd^ day, rats from different groups showed no significant difference in mitochondrial respiratory chain complex II activity (*P* > 0.05); in comparison to the control group, mitochondrial respiratory chain complex I, III, and IV activities significantly declined in CUMS group (*P* < 0.05,* P* < 0.01,* P* < 0.05); in comparison to the CUMS group, treatment with XPKYF at 14.4g/kg could suppress the decline of complex III activity (*P* < 0.05) (Table 4).

### 3.6. Effects of XPKYF on Mitochondrial Ultrastructure

**Figure 8:**
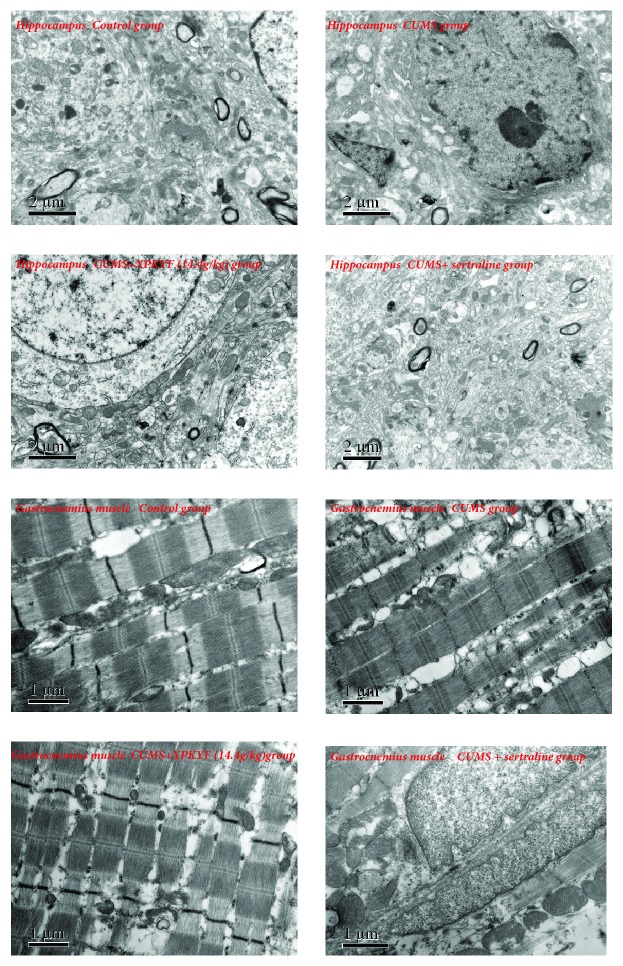
Hippocampus and gastrocnemius muscle mitochondrial ultrastructure. CUMS paradigm damaged mitochondrial ultrastructure of hippocampus tissue and gastrocnemius muscle. Treatment with XPKYF or sertraline could prevent the damage of mitochondrial ultrastructure. n=3 rats per group.

At the 14^th^ day and the 42^nd^ day, we conducted the ultrastructure observation of mitochondria. Through TEM images, compared with the control group, obvious ultrastructural changes of mitochondria were found in CUMS group at the 42^nd^ day. However, no significant ultrastructural changes of mitochondria among groups at the 14^th^ day were found in hippocampus and gastrocnemius muscle tissues. As follows, the swelling mitochondria markedly increased and the cristae disorder and mitochondrial membrane breach occurred in CUMS group of hippocampus and gastrocnemius muscle tissues. In addition, disorderly arranged myofibrils, unclear sarcomeres, and fractured myofibrils were observed in gastrocnemius muscle tissue. To some extent, treatment with XPKYF or sertraline could inhibit these ultrastructural changes of mitochondrial damage and the impairment of myofibrils and sarcomeres (Figure 8).

## 4. Discussion

In present study, rats subjected to six weeks of CUMS exhibited decreased sucrose preference ratio and prolonged immobility time. Furthermore, in hippocampus, CUMS reduced ATP content, Na/K-ATPase activity, respiratory chain complex I, III, and IV activities, and impaired mitochondrial ultrastructure; in gastrocnemius muscle, CUMS reduced Na/K-ATPase activity, respiratory chain complex I, III, and IV activities, and impaired mitochondrial ultrastructure. Rats in group treatment with XPKYF at 14.4g/kg, the efficacy trend of which was better than the other drug groups, could prevent the stress-induced depressed behavior changes, decreased Na/K-ATPase activity of hippocampus, decreased respiratory chain complex III activities of hippocampus and gastrocnemius muscle, and protect mitochondria from structural damage which is characterized by swelling, vacuolization, matrix maldistribution, and even disorderly arrangement of mitochondrial inner cristae.

ATP is the direct energy source of neuron electrical activity and of skeletal muscles contraction. As we all know, depressed mood, anhedonia, and fatigue are three major symptoms of depression. The hippocampus, which is responsible for mental activity and cognitive function, and the skeletal muscle, which supervises motor function, are highly susceptible to ATP levels. In present study, we found that hippocampus ATP content decreased significantly in depressed rat. Compared with nondepressed rats, muscle tissue ATP content in depressed rats also has an obvious decrease trend. This present data indicated that insufficient ATP content of depressed rats and then provides another experimental evidence for the pathogenesis hypothesis of energy metabolism disorder in depression.

Na/K-ATPase is a P-type ATPase vital for cell physiological function. For example, it can establish and maintain high K^+^ and low Na^+^ concentrations in the cytoplasm to sustain ionic homeostasis [15], and it is also responsible for assembly of multiple protein complexes which allows it to perform nonpumping function such as signal transduction [24]. Up to 30% of cellular ATP expenditure is used for maintaining the Na/K-ATPase activity in a resting cell, and the inhibition of Na/K-ATPase activity caused by deficiency in energy supply is harmful for cell survival [25]. Similar to ATP content changes, significantly decreased Na/K-ATPase activities of hippocampus and gastrocnemius muscle are obviously observed in CUMS group in present study. Treatment with XPKYF at 3.6g/kg and 14.4g/kg can prevent the stress-induced decreased Na/K-ATPase activity of hippocampus. In view of multiple crucial biological function of Na/K-ATPase, stress-induced Na/K-ATPase activity changes may constitute an indispensable pathological link of depression, and XPKYF appeared to have a protective effect via preventing the decrease of Na/K-ATPase activity.

Mitochondria can produce more than 95% of cellular ATP through chemiosmotic coupling of the electron transport chain (ETC) to oxidative phosphorylation (OXPHOS). The ATP production in mitochondria requires four enzyme complexes, ATP synthase, numerous assembly proteins, and two electron carriers. Together, the complexes and electron carriers transfer electrons from NADH and FADH_2_ to molecular oxygen, and, in the meanwhile, protons are pumped across the mitochondrial inner membrane to create a proton electrochemical gradient that is required for ATP synthase to produce ATP [26]. Declines of these respiratory chain complexes activities result in cellular energy deficiency and dysfunction, especially in organs with high energy demand like muscle and brain [27]. In living organisms, there are two mitochondrial respiratory chains. One is the primary respiratory chain, which is called “NADH respiratory chain”, composed of complexes I, III, and IV. The other is the secondary respiratory chain, which is called “FADH2 respiratory chain”, composed of complexes II, III, and IV. In our study, CUMS reduced respiratory chain complex I, III, and IV activities and treatment with XPKYF at 14.4g/kg could prevent the stress-induced declines of respiratory chain complex III activities in hippocampus and gastrocnemius muscle. It is noteworthy that the biochemical pattern of genetically distinct complex III defects was often a combined respiratory chain deficiency, and the presence of assembled complex III is required for the stability or assembly of complexes I and IV [28], which partly explains the synchronous decline of complex I, III, and IV activity. The protective effects of XPKYF may be partly achieved by its influence on complex III activities in hippocampus and gastrocnemius muscle.

Mitochondrion is a closed cystic structure composed of inner membrane, outer membrane, intermembrane space, and mitochondrial matrix. The electron transport chain, embedded in the mitochondrial inner membrane, can transfer electrons derived from metabolism process to oxygen, then generate water, and release energy. The integrity of mitochondrial structure is crucial for its normal function. The intermediate metabolites of carbohydrate, fat, and amino acids are finally oxidized and decomposed via tricarboxylic acid cycle that occurred in the mitochondrial matrix. During the process of oxidation decomposition, two kinds of highly reductive electron carriers, NADH and FADH2, are produced. In aerobic conditions, O_2_ was reduced to H_2_O by electron transfer of respiratory chain in the mitochondrial inner membrane and energy was released to synthesize ATP at the same time. Present study showed that the damaged mitochondria, which are characterized by swelling, vacuolization, matrix maldistribution, and even disorderly arrangement of mitochondrial inner cristae, were increased in the hippocampus and gastrocnemius muscle of CUMS-treated rats. Treatment with XPKYF or sertraline has an anti-damage trend effect, especially XPKYF at 14.4g/kg. It suggests the antidepressant effect of XPKYF may be associated with the protection of mitochondrial ultrastructure.

Consistent with our study, other previous studies supported the mitochondrial malfunction in depression. For instance, Yu Gong et al. [29] reported that chronic mild stress inhibited mitochondrial oxidative phosphorylation and damaged mitochondrial ultrastructure in various brain regions including hippocampus, cortex, and hypothalamus of mice. Rezin et al. [30] reported that complex I, III, and IV activities were inhibited in cerebral cortex and cerebellum of depressed model rats, and complex II was not affected. However, Yu Gong and Rezin' s researches focused on central nervous system changes, lack of the data of peripheral tissues, and the intervening effects of antidepressant agents. Additionally, Rezin' s research did not show stress-related alteration of mitochondrial respiratory complexes activity in hippocampus, the reason for these disparate results on complexes might lie in the variety in stress schedule as well as stimuli intensity. The data got in gastrocnemius muscle in present study made up for the deficiency of the existing previous researches and indicated that the occurrence of depression may be related to the mitochondrial damage of multiple tissues, not only confined to the central nervous system, but also related to the peripheral system.

Learning and memory impairment are important components of multisystemic manifestations in depression, which play an important role in the development and complete recurrence of depression [31, 32]. In our previous study, the antidepressant effect of XPJY decoction is partly achieved by the improvement effect on learning and memory and by the upregulation of cAMP-PKA-CREB-BDNF signaling pathway [11]. However, it is still difficult to comprehensively understand the causes of complex multisystemic symptoms presented in depression. Mitochondria are commonly distributed in the cells of various organs and tissues, and their ubiquitous characteristics determine their important role in life activities. In recent years, more and more studies have shown that energy deficiency and mitochondrial damage may also be important pathological links of depression [7–9]. These findings provide a new perspective for further study the onset of depression, especially the core symptoms of depression such as extreme fatigue and energy loss. The present study again revealed the pathogenesis of depression from the perspective of energy levels and mitochondria damage.

It should be noted that there are still many aspects which need to be further elucidated. Apart from mitochondrial energy metabolism dysfunction, there are many other related pathological mechanisms of depression such as neurobiochemical changes, neuroendocrine, inflammation, intestinal flora imbalance, neurotrophic imbalance, oxidation stress injury, and so forth. How is the relationship between different mechanisms in the onset or the development of depression? Whether XPKYF can affect these pathological processes or not? Besides that, for multiple complex ingredients in XPKYF, the precise multiple targets effective mechanisms of XPKYF are still unclear, and further systematic researches are needed.

## 5. Conclusions

The present study has shown the central and peripheral energy deficiency and mitochondrial ultrastructure damage in a rat model of depression established by CUMS. Furthermore, the antidepressant effect of XPKYF was partly achieved through preventing stress-induced decreased Na/K-ATPase activity of hippocampus, decreasing respiratory chain complex III activity of hippocampus and gastrocnemius muscle, and protecting mitochondria from structural damage. This provides another experimental evidence for application of XPKYF in depression.

## Figures and Tables

**Table 1:** Schedule of stressors used in 42 days of CUMS procedure.

Stressor	Day	Stressor	Day
Rat screech (6h) & high frequency flash (16h)	1	Rat screech (6h) & high frequency flash (16h)	22
Water deprivation & mixed breeding (22h)	2	Light irradiation & water deprivation (16h)	23
Cold water swimming (5min)	3	Fasting & mixed breeding	24
Restraint(0.5h) & rat tail with forceps clip (5min)	4	Restraint (0.5h) & rat tail with forceps clip (5min)	25
High frequency flash & water deprivation (22h)	5	Shading (16h)	26
Wet pad	6	Fasting & mixed breeding	27
Restraint (0.5h) & rat tail with forceps clip (5min)	7	Water deprivation (16h)	28
Fasting & water deprivation (16h)	8	Elevated temperature in surroundings (16h)	29
Elevated temperature in surroundings (16h)	9	Light irradiation (12h) & cold water swimming (5min)	30
Rat screech (6h) & high frequency flash (16h)	10	Tilted rat cage at 30°angle& water deprivation (16h)	31
Light irradiation &water deprivation (16h)	11	Wet pad & fasting	32
Shading (16h)	12	Mixed breeding	33
High frequency flash (16h)	13	Shading (16h)	34
Mixed breeding	14	Restraint (0.5h) & rat tail with forceps clip (5min)	35
Elevated temperature in surroundings (16h)	15	Water deprivation (16h)	36
Shading (16h) & water deprivation (16h)	16	Cold water swimming (5min)	37
Rat screech (6h) & high frequency flash (16h)	17	Rat screech (6h) & Light irradiation (12h)	38
Light irradiation (16h)	18	High frequency flash (16h)	39
Restraint (0.5h) & rat tail with forceps clip (5min)	19	Shading (16h) & water deprivation (16h)	40
Tilted rat cage at 30°angle& water deprivation (16h)	20	Tilted rat cage at 30°angle	41
Wet pad& fasting	21	Cold water swimming (5min)	42

**Table 2:** Immobility time in forced swimming test.

Groups	Immobility time (s)	
	14^th^ day	42^nd^ day
Control	52.64 ± 9.30	43.25 ± 9.14
CUMS	43.33 ± 5.46	68.00 ± 4.97^*∗∗*^
CUMS+XPKYF 3.6g/kg	44.75 ± 5.90	57.00 ± 2.65
CUMS+XPKYF 7.2g/kg	55.83 ± 4.75	43.80 ± 5.11^△△^
CUMS+XPKYF 14.4g/kg	56.92 ± 5.55	45.33 ± 1.76^△△^
CUMS+ sertraline 4.5mg/kg	56.27 ± 7.63	51.40 ± 1.60^△^

Note: the CUMS procedures were performed 30 minutes after drug administration usually by gavage between 9am and 10am. Values were presented as mean ± SEM. n=18 rats per group at the 14^th^ day; n=9 rats per group at the 42^nd^ day. ^*∗∗*^P<0.01 versus control group; ^△^P<0.05 versus CUMS group; ^△△^P<0.01 versus CUMS group.

**Table 3:** Changes of hippocampus complex I, II, III, and IV activities.

	Complex I	Complex II	Complex III	Complex IV
**14** ^**th**^ ** day**				
Control	4.86 ± 0.35	1.92 ± 0.18	0.80 ± 0.05	0.68 ± 0.07
CUMS	3.45 ± 0.31^*∗*^	1.62 ± 0.11	0.65 ± 0.05	0.52 ± 0.04
CUMS+XPKYF 3.6g/kg	4.22 ± 0.32	1.63 ± 0.11	0.72 ± 0.08	0.57 ± 0.03
CUMS+XPKYF 7.2g/kg	4.66 ± 0.23^△^	1.85 ± 0.13	0.73 ± 0.06	0.54 ± 0.06
CUMS+XPKYF 14.4g/kg	4.51 ± 0.27	1.76 ± 0.17	0.74 ± 0.06	0.58 ± 0.04
CUMS+ sertraline 4.5mg/kg	5.00 ± 0.35^△△^	1.79 ± 0.13	0.75 ± 0.07	0.56 ± 0.04
**42** ^**nd**^ ** day**				
Control	5.02 ± 0.39	1.81 ± 0.16	0.83 ± 0.09	0.70 ± 0.09
CUMS	3.16 ± 0.26^*∗*^	1.68 ± 0.13	0.55 ± 0.04^*∗*^	0.41 ± 0.07^*∗*^
CUMS+XPKYF 3.6g/kg	4.30 ± 0.38	1.78 ± 0.16	0.76 ± 0.07^△^	0.59 ± 0.10
CUMS+XPKYF 7.2g/kg	4.40 ± 0.50	1.70 ± 0.17	0.62 ± 0.03	0.57 ± 0.06
CUMS+XPKYF 14.4g/kg	4.27 ± 0.49	1.79 ± 0.15	0.78 ± 0.07^△^	0.64 ± 0.04
CUMS+ sertraline 4.5mg/kg	4.15 ± 0.19	1.73 ± 0.16	0.67 ± 0.03	0.66 ± 0.07

Note: the CUMS procedures were performed 30 minutes after drug administration usually by gavage between 9am and 10am. Values were presented as mean ± SEM. n=6 rats per group. ^*∗*^P<0.05 versus control group; ^△^P<0.05 versus CUMS group; ^△△^P<0.01 versus CUMS group.

**Table 4:** Changes of gastrocnemius muscle complex I, II, III, and IV activities.

	Complex I	Complex II	Complex III	Complex IV
**14** ^**th**^ ** day**				
Control	3.36 ± 0.30	1.69 ± 0.15	0.68 ± 0.08	0.51 ± 0.05
CUMS	2.65 ± 0.16	1.62 ± 0.18	0.52 ± 0.03	0.41 ± 0.05
CUMS+XPKYF 3.6g/kg	3.02 ± 0.30	1.54 ± 0.15	0.60 ± 0.06	0.45 ± 0.04
CUMS+XPKYF 7.2g/kg	2.88 ± 0.30	1.34 ± 0.07	0.55 ± 0.05	0.44 ± 0.05
CUMS+XPKYF 14.4g/kg	2.85 ± 0.29	1.45 ± 0.13	0.56 ± 0.03	0.50 ± 0.05
CUMS+ sertraline 4.5mg/kg	2.87 ± 0.23	1.42 ± 0.07	0.58 ± 0.06	0.48 ± 0.04
**42** ^**nd**^ ** day**				
Control	3.73 ± 0.41	1.99 ± 0.04	0.73 ± 0.07	0.55 ± 0.08
CUMS	2.36 ± 0.20^*∗*^	1.41 ± 0.17	0.42 ± 0.02^*∗∗*^	0.33 ± 0.03^*∗*^
CUMS+XPKYF 3.6g/kg	3.00 ± 0.26	1.58 ± 0.15	0.61 ± 0.06	0.43 ± 0.03
CUMS+XPKYF 7.2g/kg	2.75 ± 0.29	1.56 ± 0.21	0.58 ± 0.06	0.39 ± 0.05
CUMS+XPKYF 14.4g/kg	3.32 ± 0.28	1.67 ± 0.17	0.64 ± 0.06^△^	0.47 ± 0.05
CUMS+ sertraline 4.5mg/kg	3.02 ± 0.27	1.57 ± 0.21	0.60 ± 0.06	0.44 ± 0.07

Note: the CUMS procedures were performed 30 minutes after drug administration usually by gavage between 9am and 10am. Values were presented as mean ± SEM. n=6 rats per group. ^*∗*^P<0.05 versus control group; ^*∗∗*^P<0.01 versus control group; ^△^P<0.05 versus CUMS group.

## Data Availability

The data used to support the findings of this study are available from the corresponding author upon request.
